# Challenges in Diagnosis and Management of Spondylodiscitis of L-5/S-1 Due to *Propionibacterium acnes* in a Patient with a Twenty-Year History of Tetraplegia

**DOI:** 10.3390/clinpract16060106

**Published:** 2026-05-31

**Authors:** Vaidyanathan Subramanian, Bakulesh Madhusudan Soni, Peter Lyndon Hughes, Tun Oo

**Affiliations:** 1 Northwest Regional Spinal Injuries Centre, District General Hospital, Town Lane, Southport PR8 6PN, Merseyside, UK; bakul.soni@merseywestlancs.nhs.uk (B.M.S.);; 2 Formerly, Department of Radiology, District General Hospital, Town Lane, Southport PR8 6PN, Merseyside, UK

**Keywords:** spinal cord injury, spondylodiscitis, *Propionibacterium acnes*, shared decision-making, autonomic dysreflexia, tetraplegia, hospital to community, active surveillance

## Abstract

**Objectives:** To present novel strategies in the diagnosis and management of spondylodiscitis in a patient with tetraplegia. **Case Presentation:** A 44-year-old man presented with increased spasms despite having a SynchroMed II Medtronic for intrathecal infusion of baclofen. The C-reactive protein level was raised. Infection was suspected, but there were no localising signs. Positron Emission Tomography (PET) was performed to identify the focus of infection. PET showed an increased Fluorodeoxyglucose uptake in the L-5 vertebral body and the associated ill-defined soft tissue anteriorly. There was significant erosion and destruction of the S-1 vertebra. Magnetic Resonance Imaging of the lumbo-sacral spine revealed destruction and collapse of the S-1 vertebral body and a 6 cm × 2 cm anterior paravertebral abscess collection. This patient was managed in his home. Intravenous administration of 1 g of Ertapenem daily was provided by community nurses for eight weeks. Blood tests were performed in the community setting, and the patient was monitored by spinal unit doctors. Results: Follow-up CT revealed abnormal soft tissue, expanding and replacing the S-1 vertebral body, with appearances in keeping with an infective process. Using CT guidance and the Madison bone biopsy kit, multiple cores were obtained from the left sacral bone. Four of the five specimens showed no growth after extended incubation. *Propionibacterium acnes* were isolated after 10 days of incubation from the tissue from the sacral bone biopsy only. A shared decision was made towards active surveillance. Follow-up CT of the abdomen showed a stable appearance of the lumbar and sacral spine. **Conclusions:** A complex case of spondylodiscitis can be diagnosed and managed while the patient stays mainly in their home, avoiding prolonged admission to the spinal unit, in alignment with the “hospital to community” aspect of the National Health Service’s 10-year Health Plan for England. A diagnostic pathway with PET-CT as the first approach proved useful when the site of infection was unclear. Active surveillance obviated the need for extended periods of antibiotic therapy, which could have led to complications such as antibiotic-induced toxicity and microbial resistance to antibiotics.

## 1. Introduction

The National Health Service’s 10-year Health Plan for England states that “We will reinvent the National Health Service through 3 radical shifts: ‘hospital to community’, ‘analogue to digital’, and ‘sickness to prevention’” [1]. At its core, the Neighbourhood Health Service will embody the preventative principle that care should happen as locally as it can: in a patient’s home if possible, in a neighbourhood health centre when needed, and in a hospital if necessary.

We present the case of a male patient with tetraplegia who developed spondylodiscitis of L-5/S-1 and was managed mostly in his home as per his wishes. This concept requires a change in mindset. The delivery of care was aided by holding meetings between doctors and health professionals in several specialties over Microsoft Teams and frequent communication via email. A highly secure, encrypted email service designed to protect sensitive patient data, compliant with National Health Service security standards, was available to ensure confidentiality.

Several new management strategies were implemented for this patient. The novelties in this case are:

   i. An outpatient/community management model that avoided prolonged hospitalisation and its associated complications.
  ii. A diagnostic pathway with PET-CT as the first approach when the site of infection was unclear.
 iii. Choice of antibiotic based on well-thought-out clinical reasoning.
 iv. Administration of intravenous antibiotics for eight weeks by District Nurses in the home of a cervical spinal cord injury patient.
  v. Paucibacillary infection state achieved with *Propionibacterium acnes* with the eight-week course of antibiotics, as shown by bone biopsies.
 vi. Active surveillance instead of prolonged antibiotic therapy, resulting in a satisfactory clinical outcome (Figure 1).

## 2. Case Report

A 44-year-old male patient with tetraplegia due to cervical spinal cord injury had been experiencing increased spasms despite receiving intrathecal baclofen 525 mcg/day via the implanted SynchroMed II pump. He would start sweating when he sat up, but did not develop a headache, which is a classical feature of autonomic dysreflexia. In April 2022, his level of C-reactive protein was 23 mg/L (reference range: 0–4). An ultrasound scan of the kidneys showed mild prominence of the left renal pelvis, which had remained unchanged since 2018. In July 2022, the C-reactive protein level increased to 40 mg/L. The patient was also sweating more than usual.

A follow-up ultrasound scan of the kidneys in July 2022 showed less pronounced left renal back pressure. A left renal lower calyceal stone of 6 mm was noted.

Increased spasms, raised C-reactive protein of 71 mg/L, and a white cell count of 10.2 were worrying; further investigations were carried out to look for the source of infection. The patient was smoking 10 cigarettes a day. The chest X-ray showed both lungs appearing clear, with no active lung lesions.

The patient was also feeling hot and developed episodes of autonomic dysreflexia, as manifested by goosebumps on the arms and legs.

The suprapubic urinary catheter had been draining urine correctly, and the patient did not notice any urine leak from the penis. He did not have clinical features of urine infection.

He did not have constipation or a cough. He was experiencing spasms everywhere, and his pectoral muscles were twitching.

The patient had been sweating for three months, and the degree of sweating increased as time went on. He was also sweating on the forehead, face, and back.

The X-rays of the thoracic, lumbar, and lumbo-sacral spine showed ankylosis of the thoracic spine and ankylosis of the lower lumbar spine (Figure 2).

In the absence of localising symptoms and signs, a PT CT was carried out to identify the site of infection.

Half-body Positron Emission Tomography images were acquired from the mid-brain to mid-thighs at 60 min post Fluorodeoxyglucose (FDG) injection (Figure 3). An increased FDG uptake was seen (SUV max = 6.6) in relation to the L-5 vertebral body and the associated anterior ill-defined soft tissue.

The FDG uptake can be seen to extend posteriorly, predominantly on the left side, and to involve the left L5/S1 facet joint, where there was extensive degenerative change. There was significant erosion/destruction of the S1 vertebra.

Subsequently, Magnetic Resonance Imaging of the lumbo-sacral spine revealed destruction and collapse of the S-1 vertebral body with some fluid in what remained of the collapsed vertebral body (Figure 4).

A 6 cm × 2 cm anterior paravertebral abscess collection was seen. Significant abnormal enhancing tissue was present, extending inferiorly along both paravertebral regions and around the posterior facet joints. There was a 6 cm × 2 cm abscess collection anterior to the L5/S1 vertebral segments and a further 2 cm posterior epidural abscess.

There was an abnormally high signal within the L5/S1 disc, with appearances in keeping with L5/S1 discitis and infective collapse of S1. Osteomyelitis involving L5, the remainder of S1 and S2, and paravertebral abscesses (both a quite large anterior and a smaller posterior type) caused some effacement of the cauda equina nerve roots.

The neurosurgeon judged that this patient was not a suitable candidate for surgery and advised 6 weeks of intravenous antibiotics empirically, as guided by local microbiology. Repeat imaging and inflammatory markers at completion of treatment were prescribed; if imaging appearances improved and inflammatory markers were normal, the patient could be discharged at that point.

We looked for a positive microbiology report that would guide the choice of antibiotic. A swab taken from the suprapubic cystostomy site on 6 September 2022 showed growth of *Staphylococcus aureus* and group B Streptococcus. A sample of urine taken on 6 September 2022 showed growth of *Staphylococcus aureus* and *Enterococcus*. Both these organisms are sensitive to Trimethoprim.

This patient was prescribed Trimethoprim at 200 mg twice daily for seven days from 8/9 September 2022. Following this course of antibacterials, the CRP level decreased.

Based on the chronology of events, it was possible that Staphylococcus from the urinary tract could be the source of infection causing the spinal abscess. However, we could not ignore the possible additional role played by urinary Gram-negative pathogens. We examined the reports of urine cultures taken during the past six years. Urine had grown *E. coli* (12 July 2019), *Proteus* species (6 March 2019), and coliforms (6 July 2016). Pseudomonas was never isolated from the urine. Therefore, we decided to prescribe 1 g of intravenous Ertapenem daily for six weeks. Ertapenem was likely to be effective against *Staphylococcus aureus* as well as coliforms.

We liaised with the community to provide intravenous antibiotic therapy for the administration of 1 g of Ertapenem daily for the next six weeks.

A PICC line was inserted. The patient began to do better; he felt well enough to take the dog out and did not develop any new symptoms.

The District Nurses took blood samples at regular intervals to monitor the full blood count and C-reactive protein. We requested that the nurses send the results of the blood tests to the spinal unit.

We discussed the case with the Consultant Microbiologist at the hospital and monitored progress.

On 6 September 2022, the C-reactive protein level was 126 mg/L. Blood tests completed in October 2022 showed a C-reactive protein level of 20 mg/L. The alkaline phosphatase level was 155 µ/L on 20 October 2022. The reference range was 30–130.

A follow-up MRI of the lumbo-sacral spine was performed to assess the efficacy of the antibiotic therapy (Figure 5).

An MRI was performed on 22 September 2022, showing “L5/S1 discitis, infective collapse of S1. Osteomyelitis involving L5 the remainder of S1 and S2 and paravertebral abscess quite large anteriorly and smaller more posteriorly”.

After receiving an intravenous antibiotic (Ertapenem) for eight weeks, this patient initially showed an improvement in clinical symptoms. However, the spasms came back; similarly, he started sweating when he was sat up.

Therefore, a CT scan of the thoraco-lumbo-sacral spine was performed on 17 April 2023 (Figure 6).

The CT scan revealed abnormal soft tissue expanding and replacing the S1 vertebral body. Its appearance was broadly similar to the MRI taken in December 2022 and would be in keeping with the infective process.

The C-reactive protein level was also gradually rising.

To summarise, the patient received Ertapenem on an empirical basis. He showed improvement but did not recover fully. The symptoms of sweating and increased spasms triggered by sitting upright reappeared.

We were concerned whether there was a recrudescence of the infective process around S-1 because of inadequate therapy. It was possible that the microorganism was not fully sensitive to Ertapenem to begin with, or developed resistance during therapy, or that we did not administer the correct antibiotic, as no specimen was obtained for culture and sensitivity.

The patient did not have diabetes, cancer, or inflammatory disease, e.g., rheumatoid arthritis, vasculitis, or polymyalgia rheumatica. He was not taking insulin or metformin, immunosuppressive medication, corticosteroids, or checkpoint inhibitors, nor did he have chronic kidney disease or liver failure.

Blood tests carried out on 17 April 2023:

Serum creatinine: 45 µmol/L.

Bilirubin: 4 µmol/L.

Alkaline Phosphatase: 113 µ/L.

ALT: 8 µ/L.

Glucose Random: 4.9 mmol/L.

C-reactive Protein: 39 mg/L.

White cell count: 9.1.

Neutrophils: 6.4.

Fluorodeoxyglucose PET was performed to assess the spondylodiscitis status following antibiotic therapy (Figure 7).

Fused PET/CT images show moderate to intense uptake along the periphery of known, poorly marginated soft tissue replacing the Sacral-1 vertebral body, consistent with reactive changes (Figure 7). Tracer uptake reached up to the posterior elements of Lumbar-5, suggestive of its extension. Central photopenia was likely due to internal necrosis. Sclerotic changes in the adjacent Lumbar-5 and sacral vertebral bodies were likely reactive changes.

The MRI of the lumbar and sacral spine with contrast, performed on 16 May 2023, showed disco-vertebral osteomyelitis at the L5/S1 level with destruction of S1 and a prevertebral inflammatory collection measuring 8 cm. Enhancement of the L5 vertebral and both sacral ala and medial aspect of both iliac bones was seen, secondary to the inflammation. There was an epidural collection at the level of S1 (Figure 8).

It was possible that the empirical therapy with Ertapenem did not eradicate the infection. Another course of an antibiotic prescribed without the sensitivity report could prove futile and might even cause side effects without conferring any benefit to the patient.

Therefore, the proposed strategy was to try to get a specimen from the spinal lesion for microbiological analysis. This would enable the isolation and identification of the pathogen and allow sensitivity tests to be performed against antimicrobials. We should then be able to prescribe the most appropriate antibiotic to treat the ongoing, active infective process in the lumbo-sacral spine.

Considering the patient’s tetraplegic status and evidence of autonomic dysreflexia, it was decided to take core biopsies from the left sacral bone, which demonstrated oedema and enhancement on the recent MRI. The patient gave informed consent. With the patient in a lateral position, and using an aseptic technique, 10 mls of 1% lidocaine and 10 mls 0.5% levo bupivacaine were used for local anaesthesia, with 1 g of Paracetamol given intravenously. Using CT guidance and the Madison bone biopsy kit, multiple cores were obtained from the left sacral bone, along with bone marrow aspirate (Figure 9).

The specimens were divided into six sample pots, five of which were intended for microbiological analysis, including core samples and bone marrow aspirate. Pot 6, containing two cores, was sent to histopathology. Haemostasis was achieved. The procedure was tolerated well by the patient. There were no immediate complications. The results of the microbiological analysis are given in Table 1.

Four of the five specimens showed no growth after extended incubation. *Propionibacterium acnes* were isolated after 10 days of incubation from the tissue from the sacral bone biopsy only. The benefits and risks of further courses of antibiotics or active surveillance were discussed with the patient. A shared decision was made towards active surveillance. Active surveillance is not the same as watchful waiting; it involves a proactive management strategy including a strict regime of blood tests (full blood count and C-reactive protein every month or sooner if the patient develops symptoms of autonomic dysreflexia, temperature or feeling unwell) and clinical assessment every week.

The patient continued to get better and did not require long-term antibacterial therapy or surgical drainage of the abscess. He was able to sit up on his electric wheelchair and was independent in his mobility.

The patient underwent replacement of the baclofen pump in December 2023 and was able to manage well. The spasms and sweating settled.

A follow-up CT of the abdomen was performed on 17 October 2024. This showed bony destruction of the S-1 vertebra with soft tissue component and debris with evidence of pseudo-joint formation, and diffuse bony sclerosis of the facet joints (Figure 10A). The appearance was much more stable in comparison to the CT performed on 13 June 2023 (Figure 10B), with a slight reduction in the amount of internal debris. During follow-up over the subsequent three years, the patient did not require surgical stabilisation of the affected spine, as he was able to pursue his daily life without any constraints. The patient was last seen in the spinal unit clinic in March 2026 and followed up with over the phone in May 2026; he has been doing well and attending to his business.

## 3. Discussion

This case illustrates novel approaches in management.

1. Instead of the traditional method of prolonged hospital admission with repeated courses of antibiotics, we followed an outpatient/community management model.
2. We adopted a diagnostic pathway in which the first approach of the investigation was PET-CT when the site of infection was unknown. Our patient, who had tetraplegia, showed no localising signs, and spondylodiscitis occurred in the paralysed part of the body (L-5/S-1). PET-CT imaging helped in early diagnosis and guided further investigations and treatment.
3. Following the initial course of antibiotics, a shared decision was made towards active surveillance instead of prolonged antibiotic therapy, with a satisfactory outcome.

A review of 253 patients with vertebral osteomyelitis by McHenry and associates [2] showed that an optimal outcome of vertebral osteomyelitis requires early diagnosis, prompt identification of pathogens, and prolonged antimicrobial therapy. In this case, PET-CT helped to localise the infection, and antibiotic therapy was started.

We tried to obtain a sample for microbiology from the spondylodiscitis before starting antibiotics. However, due to organisational issues, we were unable to arrange a CT-guided biopsy at that time.

We presumed that the source of infection was urine or the suprapubic cystostomy site. We reviewed the urine cultures taken during the past six years, which revealed that the urine had grown *E. coli* in a specimen taken on 12 July 2019, *Proteus* species in the specimen taken on 6 March 2019, and coliforms in a specimen taken on 6 July 2016. Pseudomonas was never isolated from the urine. Therefore, we decided to prescribe 1 g of intravenous Ertapenem daily for six weeks. Ertapenem was likely to be effective against *Staphylococcus aureus* as well as coliforms. The advantage of treatment with Ertapenem is that Ertapenem is only required to be administered once daily. In the community, once-daily antibiotic therapy is convenient for the nurses who administer the intravenous medication, the carers, and the patient, in contrast to antibiotics such as Meropenem or Piperacillin with Tazobactam, which require administration multiple times during a 24 h period. Thus, the choice of antibiotic was based on well-thought-out clinical and diagnostic reasoning. Further, the practicalities of treatment in the community were considered. Thus, real-world experience rather than an ideological approach based on theoretical knowledge helped us to formulate a plan of treatment which could be carried out successfully and, indeed, resulted in a satisfactory outcome.

Following completion of Ertapenem therapy, we managed to arrange for a CT-guided biopsy and obtained five specimens for microbiology. One of the five specimens grew *Propionibacterium acnes* on extended culture. It is likely that the Ertapenem therapy achieved near-total eradication of the bacteria, and the patient reached a stage of paucibacillary infection. This is a plausible interpretation, but the concept of paucibacillary infection remains speculative rather than proven.

*Propionibacterium acnes* is a Gram-positive bacterium that forms part of the normal flora of the skin, oral cavity, large intestine, the conjunctiva, and the external ear canal. *Propionibacterium acnes* is an opportunistic pathogen that causes a range of postoperative and device-related infections [3]. The diagnosis of *Propionibacterium acnes* infection is difficult because of its slow growth rate and low virulence. Usually, the infection manifests several months or years after an invasive procedure on the spine in an indolent fashion.

Uckay and associates [4] reviewed spondylodiscitis due to *Propionibacterium acnes* in twenty-nine cases. They stated that *Propionibacterium acnes* is one of the most frequent anaerobic pathogens found in spondylodiscitis. A documented case required (i) microbiological proof of *Propionibacterium acnes,* and (ii) clinical and radiological confirmation of inflammation in a localised region of the spine. Our case satisfied these criteria of *Propionibacterium acnes* spondylodiscitis. In a patient who has not been under antibiotic treatment, microbiological documentation requires at least two different isolates of the same *P. acnes* strain from intra-operative tissue specimens and/or abscess aspiration under radiological control. Our patient had received Ertapenem for eight weeks and therefore reached a stage of paucibacillary infection. This would explain the isolation of *Propionibacterium acnes* in only one specimen in this patient. It is possible that the detection rate of *Propionibacterium acnes* might have been greater had we employed molecular identification by polymerase chain reaction [5]. Blood macro-genomic Next-Generation Sequencing (referred to as “liquid biopsy”) may identify pathogens that traditional methods might overlook, thus obviating the necessity for expensive and invasive surgical sampling procedures. Next-Generation Sequencing identifies microorganisms by sequencing the entire genomic content of a clinical sample, thus bypassing the need for traditional pathogen culture. Nevertheless, the current absence of specific pathogen libraries for spondylodiscitis, unlike other well-established infectious sites, hampers the precise and swift detection capability of Next-Generation Sequencing in spondylodiscitis [6].

The most frequent risk factor for *Propionibacterium acnes* spondylodisciis in the series reported by Uckay and associates [4] was surgery, which was present in the history of 28 of 29 (97%) patients. The mean delay between spinal surgery and onset of disease was 34 months. Harris and associates [7] reported a patient who developed discitis seven weeks after a routine lumbar discectomy; intraoperative cultures obtained from irrigation and debridement of the disc space revealed *Propionibacterium acnes*, and appropriate intravenous antibiotic treatment was instituted. Approximately two months later, the patient showed progression to destructive osteomyelitis requiring operative stabilisation.

The slow growth rate and low virulence delay the presentation of infection due to *Propionibacterium acnes*. Mhaidli and associates [8] reported a 65-year-old man who presented with drainage at the site of instrumented spinal surgery performed 13 years ago. *Propionibacterium acnes* infection was confirmed by culture with extended incubation. Our patient did not undergo spinal surgery in its classical term, but had undergone implantation of a baclofen pump in May 2016, about six years before he presented with increased spasms and sweating. Baclofen pump implantation involves minimal spine surgery for the insertion of a catheter in the subarachnoid space, stabilising it in the paraspinal space before the catheter is connected to the pump, which is implanted in the anterior abdominal wall. We do not know whether infection with *Propionibacterium acnes* occurred during the implantation of the baclofen pump, but it remains a possibility.

This patient was managed mainly in his home following a process of shared decision-making. Shared decision-making is a collaborative two-way process in which clinicians and patients work together to make decisions about care based on the best available evidence and the patient’s informed preferences. Shared decision-making involves discussions about the options available, along with potential outcomes, benefits, harms and uncertainties [9,10]. Crucially, patient preferences, values and beliefs are considered to ensure that any decision made is the best one for the individual at that point in time.

In this case, the spinal unit doctor discussed different treatment options, risks and benefits. The patient, being aware of his preferences, personal circumstances, and values, decided the most appropriate treatment option that addressed what mattered to him. Shared decision-making reinforced the relationship between the patient and the spinal unit doctors, which was based on partnership and mutual respect. The spinal unit doctors ensured that shared decision-making always included the option of not having treatment or not changing current care.

However, the option of managing patients in their homes may not be feasible in every case. The prerequisites for treating patients with spinal cord injury and other co-morbidities in their home are as follows:

1. Patient is willing and wishes to be taken care of in the home environment.
2. The carers should be willing and able to take the patient to a local healthcare facility for tests and assessment if there is a sudden deterioration in the patient’s clinical condition.
3. The District Nurses should be willing and have adequately trained staff to take blood samples and administer antibiotics intravenously.
4. The patient’s General Practitioner and the Surgery should be happy to support the patient being cared for in the home environment.
5. The carers should be familiar with the patient’s condition, and should be able to spot any changes in the patient’s clinical situation and communicate promptly with the spinal unit doctor and the General Practitioner’s Surgery.
6. The spinal unit doctors should be able to review the patient frequently and communicate with the patient, carers, GP Surgery, and specialists in hospitals as and when required.

The benefits of managing spinal cord injury patients in their home environment include the prevention of hospital-associated deconditioning [11] and hospital-acquired infections [12]. Our patient preferred to stay in his familiar environment, i.e., his home, amidst the team of carers who were well acquainted with his care needs and daily routines.

## 4. Conclusions

1. This case illustrates the value of PET-CT imaging to identify the focus of infection when a person with spinal cord injury presents with sweating and increased spasms, and blood tests show raised C-reactive protein, while the clinical assessment fails to reveal any localising signs or symptoms.
2. Shared decision-making is of paramount importance in the management of people with spinal cord injury. The physician should discuss the benefits, risks, and alternative treatment options (including no treatment or status quo) with the patient. In this patient with tetraplegia, following shared decision-making, spondylodiscitis of L-5/S-1 was managed by antibiotic therapy followed by active surveillance instead of prolonged antibiotics.
3. Active surveillance obviated the need for extended periods of antibiotic therapy, which could have led to complications such as antibiotic-induced toxicity, microbial resistance to antibiotics, and peripherally inserted central catheter (PICC)-related venous thrombosis.
4. The concept of managing people with tetraplegia and spondylodiscitis in the community requires close collaboration with health professionals in the community and in hospitals. A dedicated team of carers made it possible to provide care in the patient’s home.

## 5. Patient’s Perspectives

“Thanks for sending this article through, very interesting read, although I must admit I didn’t understand all the medical jargon. Thankfully, we persevered in getting down to what the problem was and there was a good outcome. It was very worrying. At the time, especially not being able to isolate exactly where the infection was coming from, but we got there in the end. I’m very lucky to live in a society where we have this kind of healthcare. Otherwise, the outcome would have been very different. I do have a great team of people surrounding me with my best interests at heart. I am very lucky.”

## Figures and Tables

**Figure 1:**
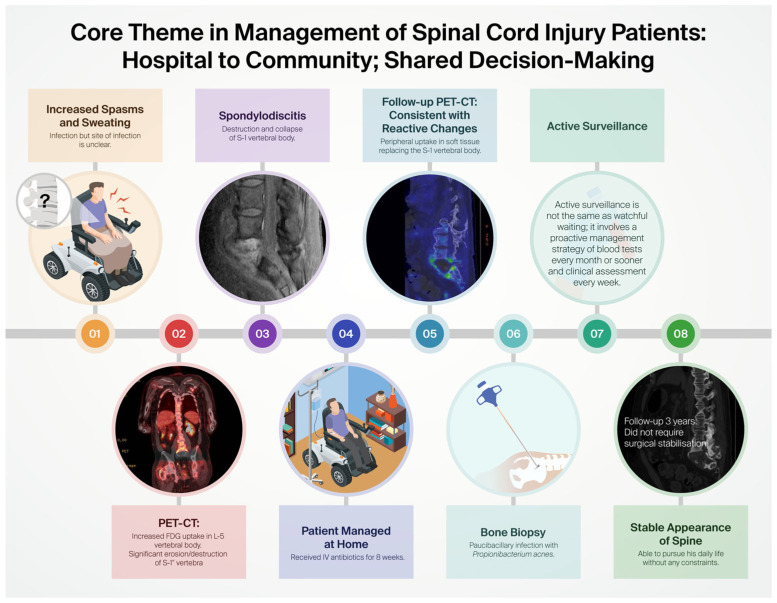
Core theme in management of spinal cord injury patients.

**Figure 2:**
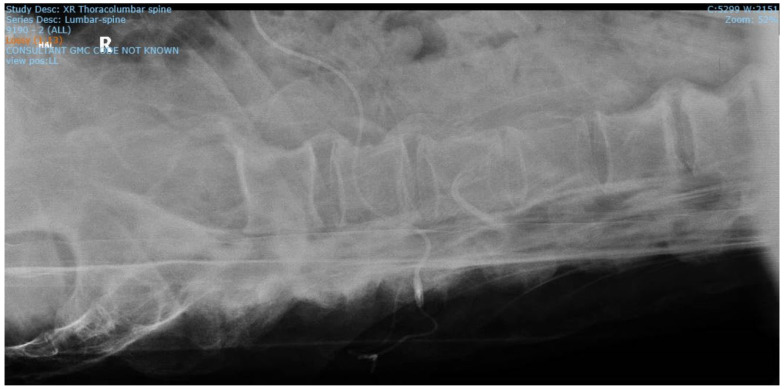
X-ray of lumbo-sacral spine (22 July 2022). The lateral view shows ankylosis of the lower lumbar spine.

**Figure 3:**
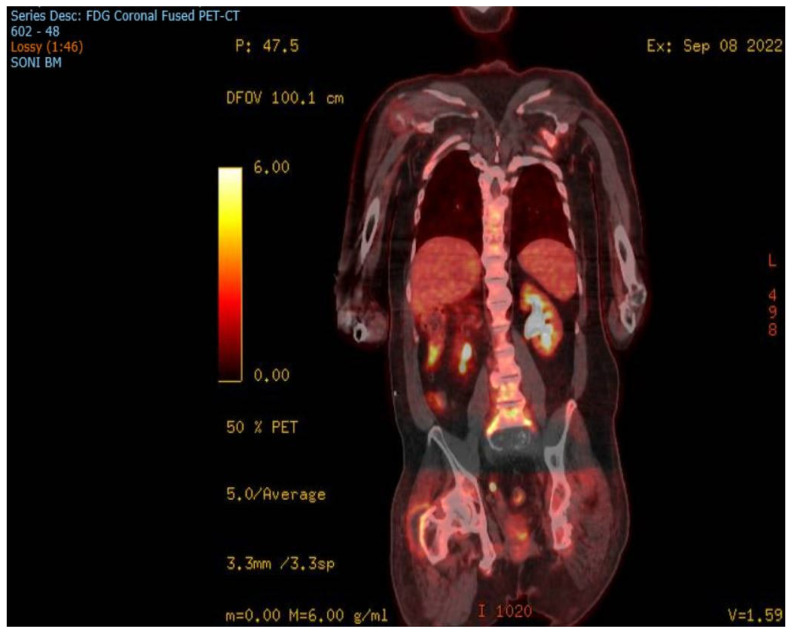
Fluorodeoxyglucose Coronal Fused Positron Emission Tomography–CT. PET image 60 min post FDG injection: coronal fused image showing increased FDG uptake (SUV max = 6.6) in relation to the L5 vertebral body and the associated ill-defined soft tissue anteriorly. The FDG uptake is seen to extend posteriorly, predominantly on the left side, to involve the left L5/S1 facet joint, where there is extensive degenerative change. There is significant erosion/destruction of the S1 vertebra.

**Figure 4:**
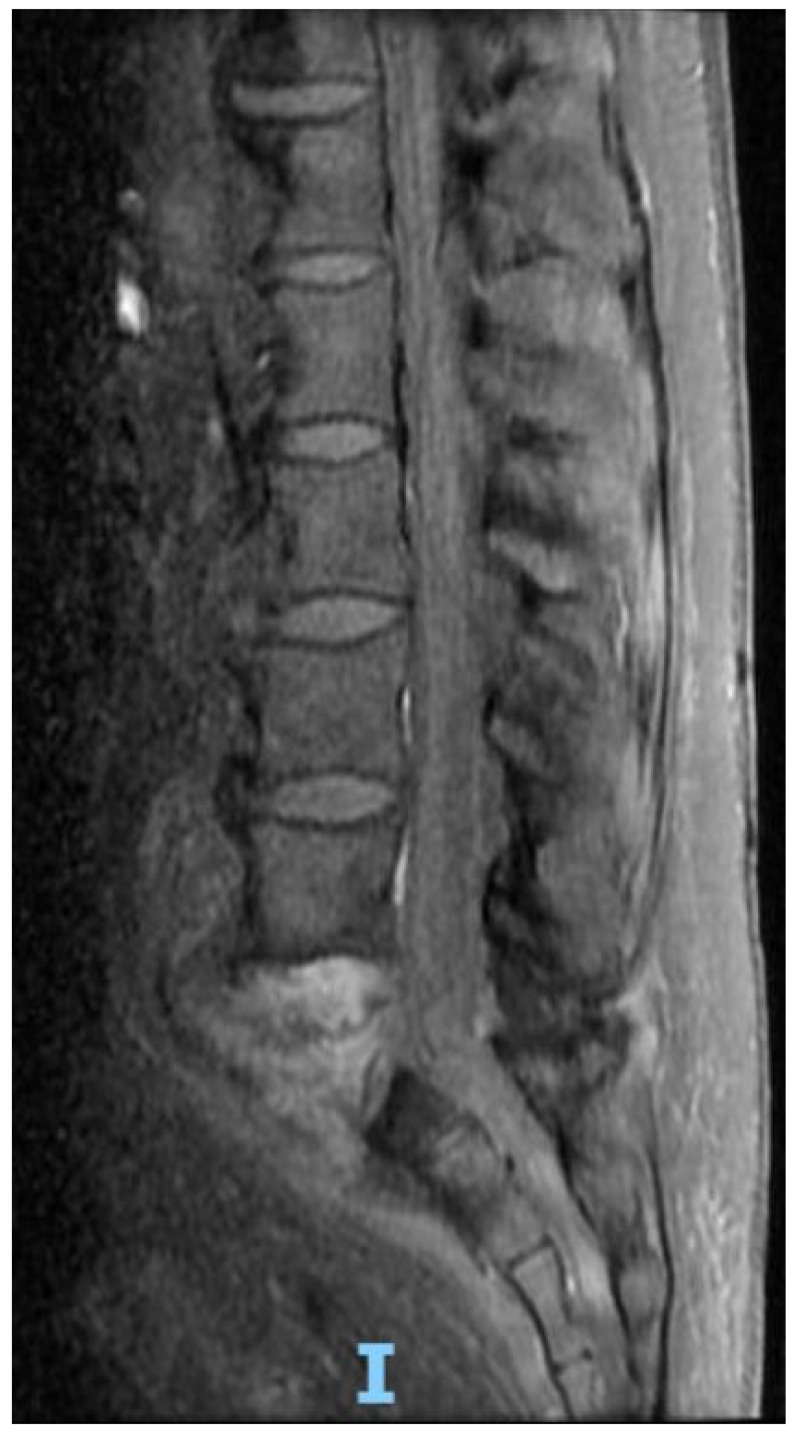
MRI of spine T-1 weighted with contrast, performed on 22 September 2022. MRI of lumbar–sacral spine with contrast T1 sagittal image (22 September 2022) shows discitis of L-5/S-1 and collapse of S-1.

**Figure 5:**
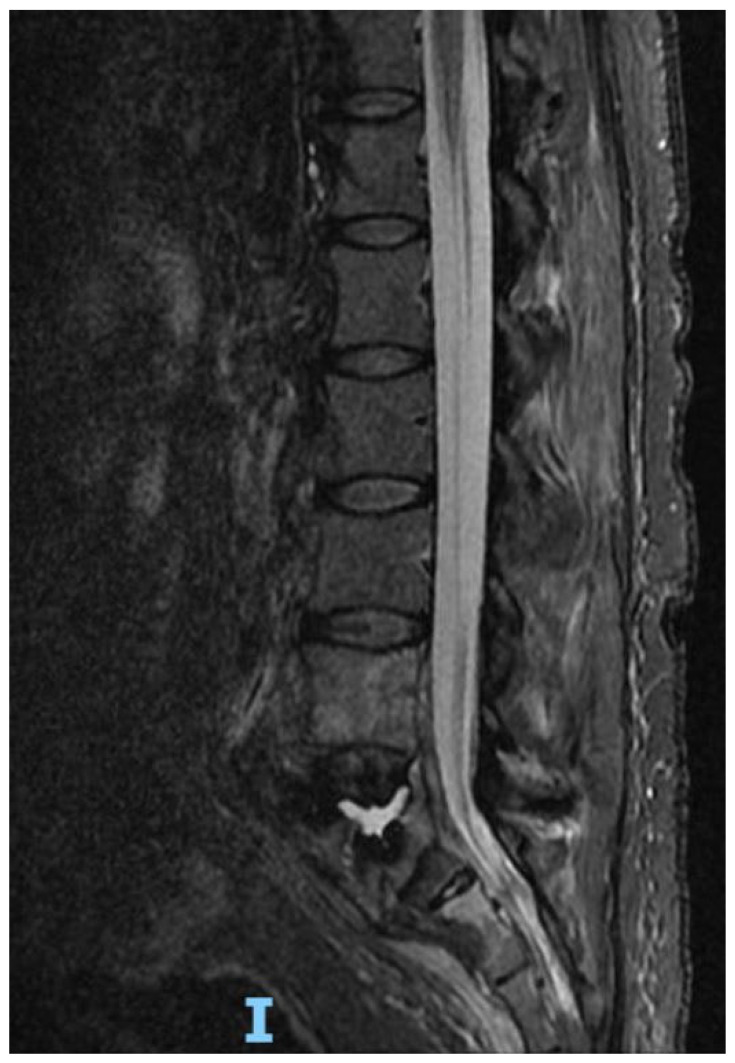
MRI of the lumbo-sacral spine: short T1 inversion recovery sequence. MRI of lumbar–sacral spine (15 December 2022) and sagittal short T1 inversion recovery (STIR) sequence showing complete collapse of S1 vertebral body. The high signal in the L5-S1 intervertebral disc is less prominent compared to the previous MRI. Some signal abnormality was again noted in the S2 and S3 vertebral bodies. The previously seen anterior collection has now been absorbed. Some scarring is seen anterior to the vertebral bodies. The spinal canal diameter is unchanged without significant compromise. Overall, there was an improvement in the radiological appearance at the L5-S1 level.

**Figure 6:**
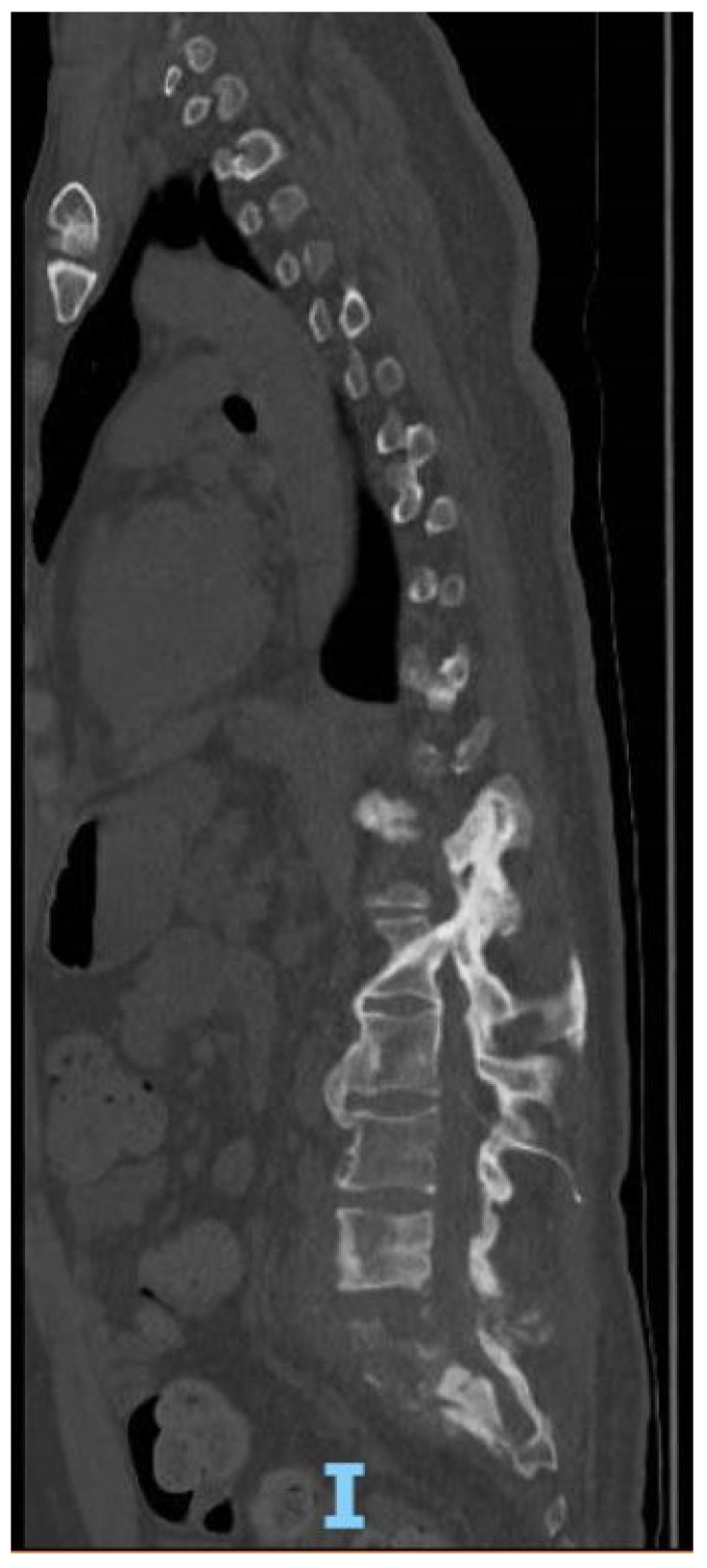
CT spine sagittal reformat on bone density. CT spine (17 April 2023) sagittal reformat on bone density showing abnormal soft tissue expanding and replacing the S-1 vertebral body. The inferior endplate of L5 appears intact. There is some sclerotic change within the L5 vertebral body. There is also patchy sclerosis of the sacrum and sclerotic change throughout the posterior elements of the lumbar and lower thoracic regions.

**Figure 7:**
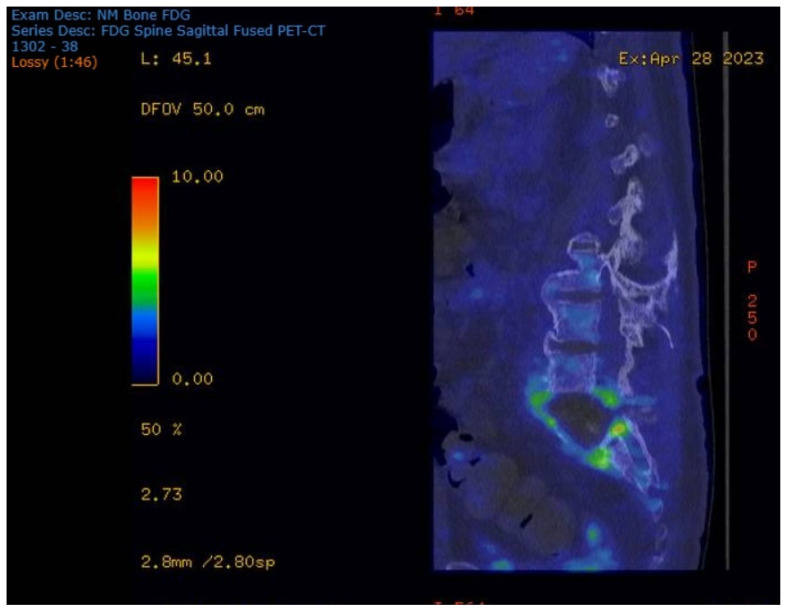
PET-CT sagittal fused image performed on 28 April 2023. Fused PET/CT sagittal image showing moderate to intense uptake along the periphery of known, poorly marginated soft tissue replacing S-1 vertebral body, consistent with reactive changes. Tracer uptake reaches up to posterior elements of L5, suggestive of its extension. Central photopenia is likely due to internal necrosis. Sclerotic changes in adjacent L-5 and sacral vertebral bodies are likely reactive.

**Figure 8:**
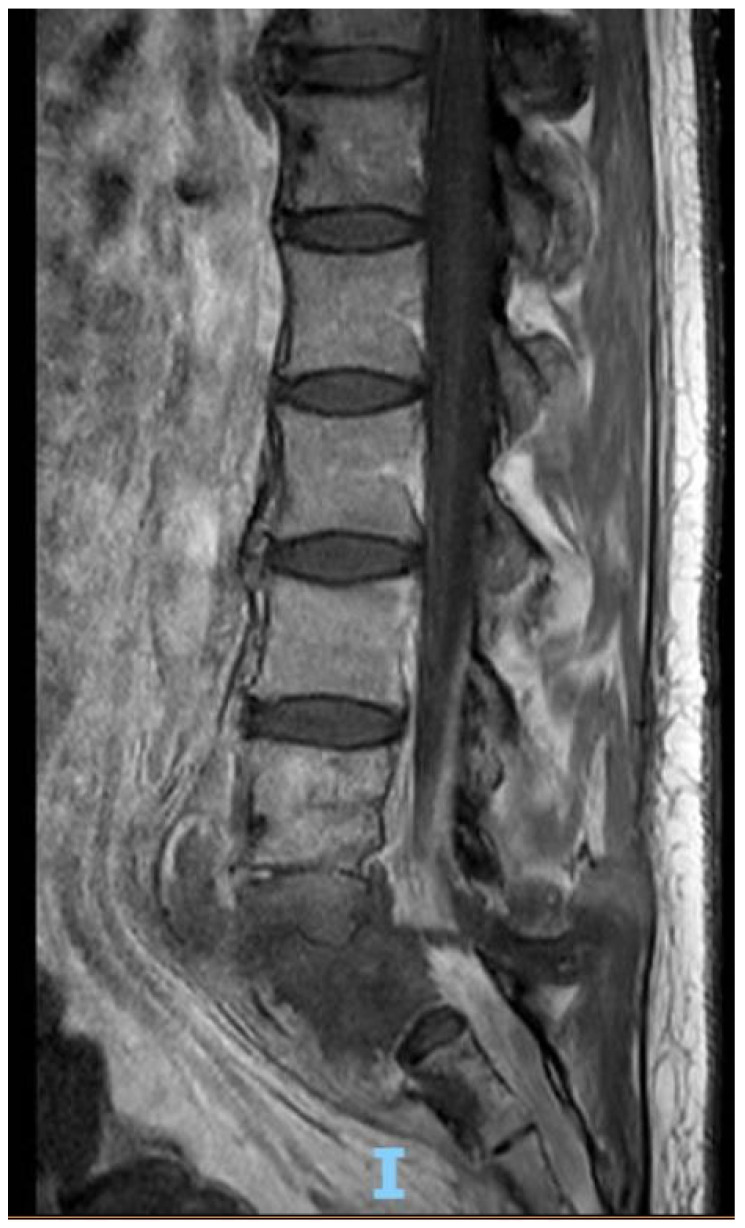
MRI of lumbar spine, sagittal T1 weighted with contrast, performed on 16 May 2023. MRI of lumbar–sacral spine with contrast. Sagittal T1 with contrast showing disco-vertebral osteomyelitis at L-5/S-1 level with destruction of S-1 and prevertebral inflammatory collection measuring 8 cm. There is enhancement of L-5 vertebral and both sacral ala and medial aspects of both iliac bones secondary to inflammation. There is epidural collection at S-1 level.

**Figure 9:**
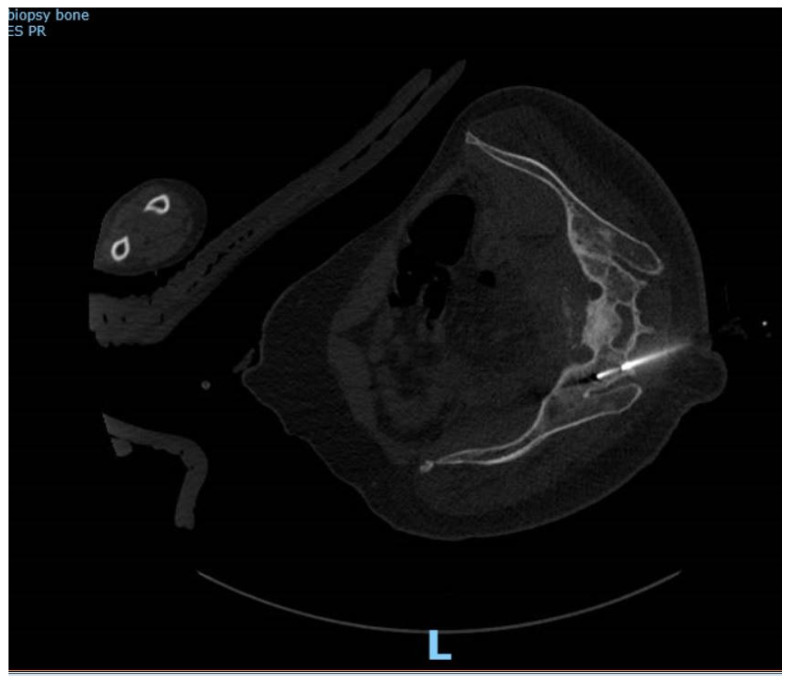
Patient in lateral position. CT-guided biopsy, pelvis on bone setting. CT-guided biopsy of bone, performed on 13 June 2023. Bone settings—patient in lateral position. Using CT guidance and Madison bone biopsy kit, multiple cores were obtained from left sacral bone along with bone marrow aspirate.

**Figure 10:**
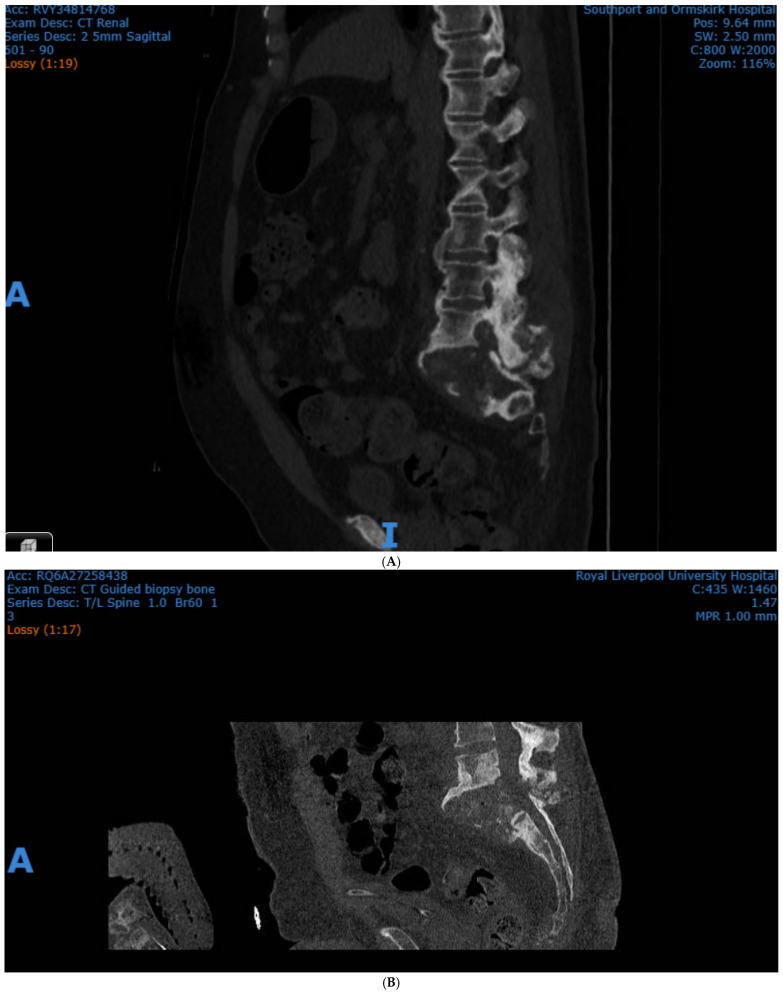
(**A**). CT of abdomen, performed on 17 October 2024. Sagittal image, bone setting. CT of abdomen, performed on 17 October 2024. Sagittal image, bone setting, showing bony destruction of the S-1 vertebra with soft tissue component and debris with evidence of pseudo-joint formation and diffuse bony sclerosis of the facet joints. Grossly stable appearance in comparison to CT scan dated 13 June 2023, with slight reduction in the amount of internal debris. (**B**). CT of lumbo-sacral spine, performed on 13 June 2023. Sagittal image, bone setting. CT scan of lumbo-sacral spine, sagittal image; bone setting shows bony destruction of S-1 vertebra, debris, pseudo-joint formation with bony sclerosis and involvement of the facets.

**Table 1:** Results of the five samples that were sent to the Microbiology Laboratory following a CT-guided biopsy performed on 13 June 2023.

Description of the Specimen	Result After 72 h of Incubation	Result After Extended Incubation
Sacral core biopsy	No growth obtained after 72 h of incubation	No growth after extended incubation
Tissue: bone marrow	No growth obtained after 72 h of incubation	No growth after extended incubation
Tissue from sacral bone biopsy	No growth obtained after 72 h of incubation	No growth after extended incubation
Tissue: no site mentioned	No growth obtained after 72 h of incubation	No growth after extended incubation
Tissue from sacral bone biopsy	Culture of the bone	*Propionibacterium acnes* isolated after 10 days of incubation

## Data Availability

The original contributions presented in this study are included in the article. Further inquiries can be directed to the corresponding author.
